# Altered sleep and neurovascular dysfunction in alpha-synucleinopathies: the perfect storm for glymphatic failure

**DOI:** 10.3389/fnagi.2023.1251755

**Published:** 2023-08-24

**Authors:** Mariateresa Buongiorno, Clara Marzal, Manel Fernandez, Natalia Cullell, Lorena de Mena, Gonzalo Sánchez-Benavides, Alejandro de la Sierra, Jerzy Krupinski, Yaroslau Compta

**Affiliations:** ^1^Hospital Universitari MútuaTerrassa/Fundacio Docència i Recerca MútuaTerrassa, Terrassa, Spain; ^2^Lab of Parkinson Disease and Other Neurodegenerative Movement Disorders, Institut d’Investigacions Biomèdiques August Pi I Sunyer (IDIBAPS), Hospital Clínic de Barcelona, Institut de Neurociències (UBNeuro), Universitat de Barcelona, Barcelona, Spain; ^3^Barcelonaβeta Brain Research Center, Barcelona, Spain; ^4^IMIM (Hospital del Mar Medical Research Institute), Barcelona, Spain; ^5^Centro de Investigación Biomédica en Red de Fragilidad y Envejecimiento Saludable (CIBERFES), Madrid, Spain; ^6^Department of Life Sciences John Dalton Building, Faculty of Science and Engineering, Manchester Metropolitan University, Manchester, United Kingdom; ^7^Parkinson’s Disease and Movement Disorders Unit, Neurology Service, Hospital Clínic i Universitari de Barcelona, CIBERNED (CB06/05/0018-ISCIII), ERN-RND, UBNeuro Institut Clínic de Neurociències (Maria de Maeztu Excellence Centre), Universitat de Barcelona, Barcelona, Spain

**Keywords:** alpha-synucleinopathies, sleep disturbances, glymphatic system, cognitive decline, Parkinson’s disease, multisystem atrophy, Lewy bodies dementia, circadian blood pressure patterns

## Abstract

Clinical and cognitive progression in alpha-synucleinopathies is highly heterogeneous. While some patients remain stable over long periods of time, other suffer early dementia or fast motor deterioration. Sleep disturbances and nocturnal blood pressure abnormalities have been identified as independent risk factors for clinical progression but a mechanistic explanation linking both aspects is lacking. We hypothesize that impaired glymphatic system might play a key role on clinical progression. Glymphatic system clears brain waste during specific sleep stages, being blood pressure the motive force that propels the interstitial fluid through brain tissue to remove protein waste. Thus, the combination of severe sleep alterations, such as REM sleep behavioral disorder, and lack of the physiological nocturnal decrease of blood pressure due to severe dysautonomia may constitute the perfect storm for glymphatic failure, causing increased abnormal protein aggregation and spreading. In Lewy body disorders (Parkinson’s disease and dementia with Lewy bodies) the increment of intraneuronal alpha-synuclein and extracellular amyloid-β would lead to cognitive deterioration, while in multisystemic atrophy, increased pathology in oligodendroglia would relate to the faster and malignant motor progression. We present a research model that may help in developing studies aiming to elucidate the role of glymphatic function and associated factors mainly in alpha-synucleinopathies, but that could be relevant also for other protein accumulation-related neurodegenerative diseases. If the model is proven to be useful could open new lines for treatments targeting glymphatic function (for example through control of nocturnal blood pressure) with the objective to ameliorate cognitive and motor progression in alpha-synucleinopathies.

## Introduction

Aggregation of misfolded alpha-synuclein (α-syn) is the neuropathological hallmark of Parkinson’s disease (PD), dementia with Lewy bodies (DLB) and multiple system atrophy (MSA). While in PD and DLB α-syn aggregates are intraneuronal (the so-called Lewy bodies and neuritis after which they are called Lewy body disorders [LBD]), MSA is characterized by oligodendroglial cytoplasmic inclusions. Hence, albeit LBD and MSA share underlying synucleinopathy, their clinical presentation largely differs, and they disclose this different cellular-type involvement (neuronal vs. glial).

Within LBD, PD and DLB can be conceptualized as extremes of the same disease spectrum, in which the Lewy pathology is initially restricted to brainstem and midbrain in PD (Braak et al., 2003) and has early neocortical and limbic involvement in DLB (McKeith et al., 2017). In accordance with this different lesion topography, the initial symptoms are predominantly motor at PD onset, while in DLB the core feature is the early cognitive impairment either preceding or quickly following motor symptoms. Indeed, the rule of more than 1 year between the diagnosis of PD and the apparition of dementia applied to differentiate DLB and PD dementia (PDD) remains arbitrary and controversial.

Although PD has traditionally been conceived as a motor disease mainly related to alterations in the basal ganglia, up to 80% of PD patients develop dementia during the progression of the disease (Hely et al., 2008). Other patients show more stable, mild, and restricted cognitive and emotional difficulties during the disease course, putatively related to a specific decreased dopaminergic function in fronto-striatal circuits. Thus, some degree of cognitive impairment seems to be a core feature of PD, mainly related to the dopamine-mediated fronto-striatal executive alterations. By contrast, the cognitive impairment in PDD has a distinctive pattern, in which temporal and posterior cortical dysfunction predominates, causing attentional, semantic verbal fluency, and visuospatial difficulties. In fact, those PD patients that display mild cognitive impairment in these latter domains are more likely to progress to dementia (Wood et al., 2016). This observation about the different cognitive profile within PD patients has been called the “dual syndrome hypothesis” (Kehagia et al., 2013), and supports the notion of the existence of additional pathophysiological mechanisms, other than the dopamine-mediated fronto-striatal alterations, involved in the etiology of dementia in PD. The most documented clinical predictors of future cognitive decline in PD are advanced age and a longer duration of motor symptoms (Hely et al., 2005; Reid et al., 2011; Cholerton et al., 2013), as well as poorer baseline cognitive performance (Compta et al., 2013; Anang et al., 2014), male sex (Anang et al., 2014; Cholerton et al., 2018), disturbances of behavior during REM sleep and the presence of greater dysautonomia (orthostatic hypotension) (Anang et al., 2014, 2017; Schrag et al., 2017). In the present manuscript we briefly review the relevant factors involved in clinical progression in alpha-synucleinopathies (dementia in LBD and faster motor worsening in MSA) and propose a model including glymphatic system as a key mechanistic explanatory aspect.

## The pathophysiology of dementia in PDD

The progressive extension of Lewy-like pathology to cortical and limbic areas has been related to cognitive impairment and dementia in PD (Hely et al., 2008). A meta-analysis of anatomical-pathological studies has observed that the density of Lewy bodies in the cerebral cortex is associated with the severity of the cognitive disorder in PD (Smith et al., 2019). In addition, several studies have suggested that AD pathology, characterized by neuritic β-amyloid plaques (Aβ) and neurofibrillary tangles of tau protein (τ), also plays a role in dementia in PD (Sabbagh et al., 2009; Compta et al., 2011; Buongiorno et al., 2017). In this vein, several studies have shown reduced CSF Aβ as predictor of cognitive worsening and risk of dementia in PD (Siderowf et al., 2010; Buongiorno et al., 2011; Compta et al., 2013). Besides, brain atrophy has been also reported as a relevant predictor. PD patients who develop dementia initially present a smaller volume of frontal gray matter (Compta et al., 2013) and hippocampus (Low et al., 2019) than patients who remain stable. In addition to AD pathology and brain atrophy, brain vascular disease has also been suggested as a relevant predictor in the development of PDD. Presence of white matter hyperintensities in MRI predicts progression to dementia in cognitively unimpaired PD (Compta et al., 2016; Chahine et al., 2019; Dadar et al., 2020), as well as cerebral amyloid angiopathy, in which amyloid proteins build up on the walls of the arteries in the brain. More recently, another mechanism related to the failure of the glymphatic system has been suggested to play a major role in the development of dementia in protein accumulation diseases.

## Glymphatic dysfunction and sleep in neurodegenerative diseases

The glymphatic system is a recently described cerebrospinal fluid (CSF) transport system that exports the excess of interstitial fluid and proteins (Iliff et al., 2012; Nedergaard and Goldman, 2020). The CSF and peripheral lymph are drained together into the venous system through the perivascular spaces created by the vascular endfeet of astrocytes (Iliff et al., 2012). The astrocytic endfeet plasma membrane is mainly occupied by the water channel protein aquaporin-4 (AQP4) (Nagelhus et al., 2004). It is known that genetic variation in *AQP4* moderates the association between sleep and amyloid-β burden (Rainey-Smith et al., 2018), and, in AD, some specific *AQP4* SNPs are related to steeper cognitive decline, whereas others relate to slower progression (Burfeind et al., 2017). Arterial pulsatility provides the motive force for CSF transit into the perivascular spaces surrounding the major arteries (Iliff et al., 2013), whereas respiration and slow vasomotion contribute to sustaining its flow (Kiviniemi et al., 2016). The glymphatic clearance system is related to circadian rhythmicity, and seems to be active only during NREM sleep (Xie et al., 2013), when there are large coupled low-frequency oscillations in neuronal activity, blood oxygenation, and CSF flow (Fultz et al., 2019). It has been proposed that the loss of quality of sleep that appears in aging (mainly with loss of NREM stage), as well as the sleep architecture disturbances observed in many neurodegenerative diseases (Nedergaard and Goldman, 2020), may potentially cause a great decline in clearance of brain waste, as the efficacy of glymphatic fluid transport correlates directly with slow-wave activity. Therefore, the loss of glymphatic function would lead to increased α-syn aggregation as recently demonstrated in murine models of PD (Zou et al., 2019; Zhang et al., 2023), and even in neuropathological studies of individuals without PD, that showed that sleep fragmentation, as measured by actigraphy, was associated with significant presence of Lewy body pathology in 269 participants of the Rush Memory and Aging Project (Sohail et al., 2017). In the same direction, recent evidence from neuroimaging studies using diffusion along perivascular spaces (DTI-ALPS) measures have demonstrated the impairment of glymphatic function in PD patients (McKnight et al., 2021; Cai et al., 2023). In line with this findings, Sundaram et al. (2019) have proposed a framework to study the failure of the glymphatic system in PD, emphasizing the role of the early sleep disturbance observed in this pathology, characterized by REM sleep behavior disorder (RBD).

## REM sleep behavior disorder in PD

RBD is a clinical disorder characterized by loss of the normal muscle atonia during the REM phase of sleep, which results in impaired suppression of movement generators and complex dream enactment behaviors (Boeve et al., 2007). Clinically, RBD represents a primary cause of sleep quality disruption. Nearly all individuals with idiopathic RBD will eventually develop PD, DLB, or MSA (Iranzo et al., 2013; St Louis and Boeve, 2017). In contrast to the abnormal motor activity observed during REM sleep in RBD patients, all other main sleep features were classically considered as preserved (Dauvilliers et al., 2018). However, recent studies found abnormal EEG features during NREM sleep in patients with RBD, such as less density of sleep spindles, altered slow oscillations morphology, and abnormal spindle/slow oscillations coordination, suggesting that RBD-related disease processes affect neuronal populations also associated with NREM sleep regulation (Sunwoo et al., 2021; Gorgoni and Galbiati, 2022). Although the RBD pathogenesis remains unclear, it seems that dysregulation of specific brainstem areas may be involved. A recent study found that patients with idiopathic RBD display neuronal dysfunction in the peripheral autonomic nervous system and locus coeruleus equivalent to patients with PD, whereas they had normal nigrostriatal dopaminergic innervation (Knudsen et al., 2018). Therefore, in RBD, which is considered a model of prodromal PD, peripheral pathology is almost similar to patients with clinical PD and dysregulation of blood pressure (BP) may be compromised earlier than supposed.

## Blood pressure and cognitive decline in PD

Arterial pulsatility is the major mechanism for clearance of brain proteins by the glymphatic system (Iliff et al., 2013). Thus, any pathology affecting arterial pulsatility may affect the glymphatic system. It is well known that cardiovascular risk factors (e.g., hypertension, hypercholesterolemia, obesity, diabetes) negatively affect cognitive abilities (Knopman et al., 2001), whereas cardiovascular fitness positively correlates with cognition in both young and old adults. Hypertension induces hypertrophy of vascular smooth muscle cells, with a stiffening of the arterial wall that reduces pulsatility. Indeed, it has been shown that glymphatic function is suppressed in hypertensive rats (Mortensen et al., 2019).

Some studies have partially addressed the relationship between blood pressure alterations in PD and cognitive impairment. In a cross-sectional study, Kim et al. (2012) found that cognitive impairment was associated with orthostatic hypotension (OH) and supine hypertension (SH) in a mixed sample of cognitively unimpaired PD, PD-MCI and PDD, and that all patients having both alterations presented cognitive impairment. Interestingly, posterior longitudinal studies found a 3-fold increased risk of cognitive impairment in PD patients having OH (Anang et al., 2014). A review on the topic (Pilotto et al., 2019) concluded that OH and RBD yielded individual and combined negative effects on disability in α-synucleinopathies, probably reflecting a “malignant” phenotype of PD with early cognitive impairment and postural instability. Similarly, Anang et al. (2014) suggested that non-motor and “non-dopaminergic” features of PD, including autonomic dysfunction and RBD, can strongly predict development of dementia in PD.

## Nocturnal blood pressure profile and cognitive decline

Further than global BP abnormalities, aberrant nocturnal blood pressure profiles (characterized by either a blunted, less than 10%, nocturnal BP decline, namely, non-dipping profile, or an increase in nocturnal BP, with respect to daytime BP, namely riser profile), have been associated with the incidence of mild cognitive impairment in elderly population (Guo et al., 2010). The abnormalities in the circadian rhythm of BP in PD have been described decades ago (Senard et al., 1992) and, more recently, Tanaka et al. (2018) found that the nocturnal riser profile was cross-sectionally associated with the presence of dementia (odds ratio 11.6), while the physiological dipper pattern (a reduction in nighttime BP greater than 10% with respect to daytime BP) was almost inexistent in the demented group (<4%). Similarly, non-dipper PD patients also show higher degree of psychotic symptoms (Stuebner et al., 2015). Non-dipping and riser profiles seem independent of age, gender, disease duration, age at onset, the severity of the disease, the use of anti-PD drugs and vitamin D level (Arici Duz and Helvaci Yilmaz, 2020).

## Prominent dysautonomia and preserved cognition in MSA

The other major condition in the category of synucleinopathies is MSA. It clinically presents as either levodopa-unresponsive parkinsonism or cerebellar syndrome, but autonomic failure is mandatory for the diagnosis (Wenning et al., 2022). Unlike PD, pathologically misfolded α-synuclein accumulates as cytoplasmic inclusions in the oligodendrocytes. According to the conceptual framework presented in the above paragraphs, it could be argued that the prominent dysautonomia of MSA would lead to prominent cognitive impairment, but this is not the case. Although recent research has found some degree of cognitive impairment in MSA patients (Stankovic et al., 2014), cognition is typically preserved, and the observed cognitive disturbances have been associated with the presence of intraneuronal inclusions, rather than the density of oligodendroglial pathology (Koga et al., 2017). Despite the specific tropism of different α-syn strains for neurons (in PD and DLB) and for oligodendrocytes (in MSA) (Schmitz et al., 2023), they may share a common mechanism of increased accumulation of misfolded α-synuclein related with BP alterations caused by dysautonomia. In PD the cortical spreading of Lewy bodies would lead to dementia, and in MSA the rapid subcortical accumulation would have a role in the malignant motor and dysautonomic (rather than cognitive) course of this disease. Nevertheless, the reason why despite sharing the same key protein and sleep and autonomic dysfunction PD and MSA differ in their cognitive outcome warrants further elucidation, including improving our knowledge of the interplay between glymphatic dysfunction and the different strains and aggregation kinetics of α-syn.

## Discussion

**FIGURE 1:**
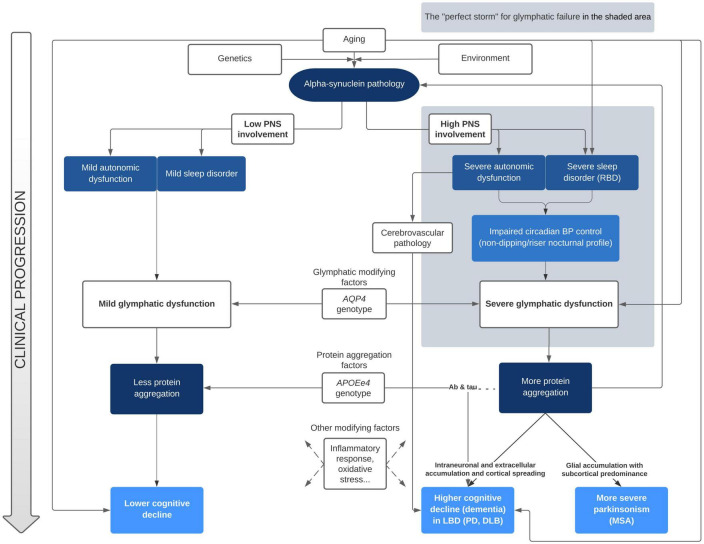
Mechanistic approach of clinical progression in alpha-synucleinopathies accounting for glymphatic function. BP, blood pressure; DLB, dementia with Lewy bodies; LBD, Lewy bodies disorders; MSA, multisystem atrophy; PD, Parkinson’s disease; PNS, peripheral nervous system; RBD, REM sleep behavior disorder.

Sleep disturbances and nocturnal BP abnormalities have been clearly identified as risk factors for cognitive deterioration in alpha-synuclein related disorders. However, to date, no research group has clearly linked both as acting through glymphatic system failure. On the one hand, Tanaka and Hattori (2022) have proposed a pathological model linking abnormal BP and cognitive decline in alpha-synucleinopathies. In their model, there are two main drivers for the acceleration of α-syn, tau, and Aβ pathology aggregation that leads to dementia: first, the diurnal BP fluctuation due to neurogenic OH, and second, the supine hypertension and the loss of the physiological nocturnal BP fall. According to their proposal, these two phenomena would produce cerebral hypoperfusion and hypoxia, leading to increased oxidative stress, neuroinflammation, augmented BBB permeability, WMH and microbleeds. Although we do not question the plausibility of the chronic hypoperfusion pathological pathway, we further propose that the malfunction of the glymphatic system plays a key role in the exacerbated abnormal protein aggregation that ultimately leads to cognitive decline. On the other hand, Sundaram et al. (2019) and Scott-Massey et al. (2022) deeply reviewed the evidence of the glymphatic failure and sleep disturbances in PD, but did not include the nocturnal BP alterations linked with dysautonomia as a mechanistic factor in their models. Our proposal linking both approaches for the first time is summarized in Figure 1.

In our view, the clinical features of patients with alpha-synucleinopathies that develop dementia conform the perfect storm for glymphatic failure. Since the glymphatic system is mainly active during deep sleep stages, it is not surprising that presence of RBD, which seems to also affect NREM sleep features, is ubiquitous in patients with rapid cognitive decline. Further, vascular pulsatility provides the motion force that propels the interstitial fluid through brain tissue to remove protein waste. Accordingly, any deviation from the physiological fall of BP that occur during the night, such as the abnormal pattern of nocturnal BP (non-dipping/riser) that is tightly related to dysautonomia, could impair the removal of waste solutes. Within this model, the paradigm of earlier and more severe glymphatic failure would be DLB, in which RBD, severe dysautonomia and early dementia is associated to widespread cortical pathology. In the other extreme of this continuum would be PD patients that during all the course of disease do not clearly deteriorate, with main symptoms restricted to the motor domain.

Our hypothesis on the major contribution of the glymphatic system failure in cognitive decline in PD, via sleep and blood pressure disturbances, also fits in the proposed *brain-first vs gut-first* hypothesis (Borghammer and Van Den Berge, 2019). According to this hypothesis, the heterogeneity observed in alpha-synucleinopathies may relate to different patterns of temporal involvement of the nervous system. While most of the patients would show a *gut-first* (i.e., peripheral-first) pattern, in which the initial pathological α-syn aggregates appear in the dorsal motor nucleus of the vagus and propagate via retrograde axonal transport (Braak et al., 2003), other patients were characterized by initial nigrostriatal dopaminergic dysfunction, without pathology in the vagus and spared autonomic function (*brain-first*). Interestingly, the proposed *gut-first* pattern is tightly related to presence of RBD, and it has been reported that patients with idiopathic RBD display neuronal dysfunction in the peripheral nervous system and locus coeruleus equivalent to PD patients, but normal nigrostriatal dopaminergic innervation (Knudsen et al., 2018). In our view, the joint mechanisms of sleep alterations and nocturnal BP dysregulation would contribute to the later worse cognitive outcome of patients with severe glymphatic failure.

Other evidence supporting the existence of this association comes from the heterogeneous clinical presentations of PD patients carrying pathogenic genetic variants. While PD patients carrying mutations of the β-glucocerebrosidase gene (GBA) have more severe dysautonomic symptoms and RBD than patients with idiopathic PD and more frequent cognitive impairment (Zhang et al., 2018; Carandina et al., 2022), PD patients with mutations in the LRRK2 gene encoding leucine-rich repeat kinase 2 (LRRK2) protein show much lower frequency of RBD (Pont-Sunyer et al., 2015; Fernández-Santiago et al., 2016), less OH (Tijero et al., 2013), and less cognitive decline (Saunders-Pullman et al., 2018; D’Souza and Rajkumar, 2020).

A possible criticism to the presented model to explain cognitive decline may relate to MSA pathology. In MSA, patients’ cognition is not typically compromised in the context of a prominent autonomic failure provoking severe BP abnormalities that may include non-dipping and rising nocturnal profiles accompanied by RBD. However, in MSA the clinical expression of severe glymphatic dysfunction via dysautonomia and sleep disturbance would be an aggressive and fast motor progression, rather than cognitive decline, owing to predominant subcortical tropism due to different strains and/or kinetics of α-syn aggregation, which might relate to pathological and clinical differences, and also to differences in seeding assays results (Compta et al., 2022; Poggiolini et al., 2022).

The presented model is agnostic regarding the *primum movens* of alpha-synucleinopathy and only focuses on the interplay between factors involved in clinical progression. It aims to foster research studies in the spectrum of alpha-synucleinopathies by incorporating nocturnal BP alterations and sleep abnormalities causing glymphatic failure as a mechanistic driver of abnormal cortical aggregation of proteins and subsequent deterioration. Adding glymphatic functioning measures, such as MRI-ALPS, circadian BP monitoring, or relevant polymorphisms, such as *AQP4*, and epigenetic biomarkers in research studies may lead to personalized risk models for dementia and the development of strategies to prevent it.

**FIGURE 2:**
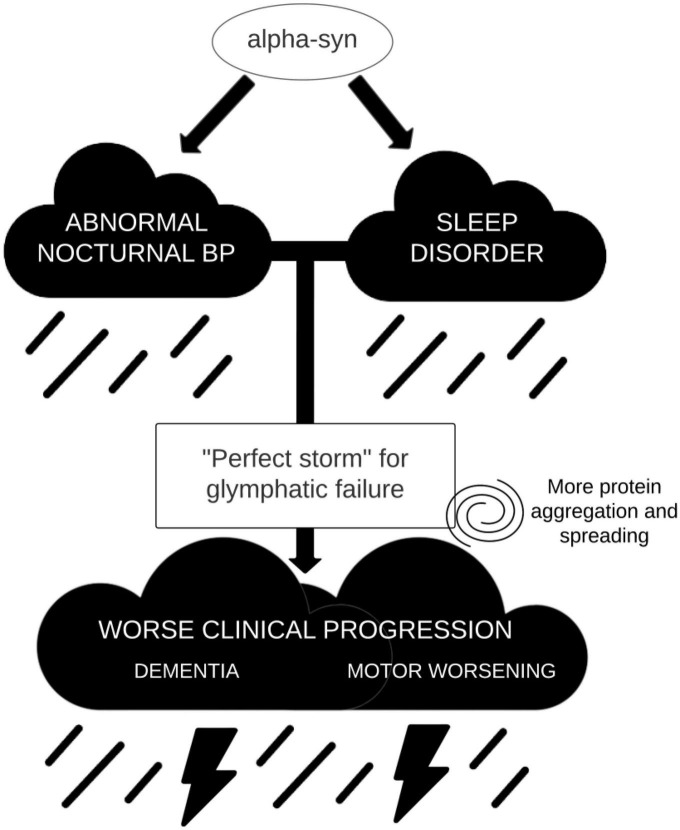
Simplified model on the combined effect of sleep disorders and nocturnal blood pressure (BP) in glymphatic failure and clinical progression.

In summary, we argue that in alpha-synucleinopathies there may be a pathophysiological link between abnormal nocturnal tension profile, associated to dysautonomia and sleep disorder, and clinical progression. Such association would be mediated by the glymphatic system functioning. Altered sleep architecture and blunted arterial pulsatility would lead to severe glymphatic failure, which in turn produces impaired waste clearance and increased abnormal protein accumulation (Figure 2). While in MSA more aggregation in glial cells would lead to more severe motor symptoms, in LBD the cortical spreading of α-syn (and other proteins such as amyloid-β) would produce dementia. If these associations could be demonstrated, we would be able to develop not only simple biomarkers to identify those patients with LBD that are more likely to develop dementia, but also, and more importantly, a research line to prevent cognitive decline in these patients, by trying to correct early in the disease the nocturnal tension profiles that are deleterious to the functioning of the glymphatic system.

## Data availability statement

The original contributions presented in this study are included in the article/supplementary material, further inquiries can be directed to the corresponding authors.

## Author contributions

MB and YC conceptualized the manuscript. MB and GS-B wrote the first draft. YC, CM, MF, NC, LM, AS, and JK critically revised the manuscript and made relevant changes. All authors approved the final version of the manuscript.
